# Amyloid-targeting treatment in Alzheimer's disease and concomitant antiplatelets: a clinical gray zone?

**DOI:** 10.3389/fneur.2026.1814289

**Published:** 2026-05-21

**Authors:** Polona Rus Prelog, Matija Zupan, Alenka Kovačič, Senta Frol, Milica Gregorič Kramberger

**Affiliations:** 1 Centre for Clinical Psychiatry, University Psychiatric Clinic Ljubljana, Ljubljana, Slovenia; 2 Faculty of Medicine, University of Ljubljana, Ljubljana, Slovenia; 3 Department of Vascular Neurology, University Medical Centre Ljubljana, Ljubljana, Slovenia; 4 General Hospital Murska Sobota, Murska Sobota, Slovenia; 5 Department of Neurology, University Medical Centre Ljubljana, Ljubljana, Slovenia; 6 Department of Neurobiology, Care Sciences and Society (NVS), Division of Clinical Geriatrics, Karolinska Institutet, Huddinge, Sweden

**Keywords:** Alzheimer's disease, amyloid-related imaging abnormalities (ARIA), anti-amyloid monoclonal antibodies, antidepressants, bleeding risk, cerebral amyloid angiopathy (CAA), herbal and dietary supplements, polypharmacy

## 1 Background: eligibility and risk considerations for amyloid-targeting therapies

Population studies that applied trial-like criteria to community cohorts estimate about 2.2 million in the US and ~5.9 million patients in the EU with mild cognitive impairment or mild dementia due to Alzheimer's disease (AD) could be eligible for amyloid-targeting treatment (ATT) (1). Apart from determining the likelihood of benefit, the key task when selecting patients for ATTs is determining the risk of adverse effects, primarily amyloid-related imaging abnormalities (ARIAs), which manifest as edema (ARIA-E) or hemorrhage (ARIA-H) on brain MRI (2–5). Since we are still in the early days of ATTs with an obvious lack of real-world data, the strict selection criteria used in seminal randomized controlled trials (RCTs) of ATTs [Clarity AD for lecanemab (6) and TRAILBLAZER-ALZ2 for donanemab (7)] limit generalizability beyond the populations studied, causing uncertainty about safety in many real-world scenarios, such as concomitant antithrombotic treatment or eligibility for thrombolytic therapy in the context of acute ischemic stroke (AIS).

ARIA shares clinical and pathophysiologic features with cerebral amyloid angiopathy (CAA), including vascular amyloid deposition and inflammatory activation. A major driver of ARIA risk is APOE ε4 carriage of the apolipoprotein E gene (homozygous individuals showing the highest risk), which is also a well-known genetic risk factor for development of AD (5, 7). The presence of CAA risk factors or CAA neuroimaging features at baseline (e.g., >4 microhemorrhages, any macrohemorrhage, vasogenic edema, CAA-related inflammation, cortical superficial siderosis), in addition to extensive white matter hyperintensities on fluid-attenuated inversion recovery MRI sequences (i.e., leukoaraiosis with Fazekas score ≥3), believed to represent severe microvascular disease, are hypothesized to correlate with the risk of developing ARIAs (6, 7). Additionally, poorly controlled bleeding disorders, especially those with a platelet count < 50,000/μL or an international normalized ratio >1.5 may enhance the risk of ARIAs and these patients were consequently excluded from the seminal RCTs (6, 7). Moreover, patients with systemic autoimmune conditions were excluded from these trials given their potential interaction with immunologic mechanisms in ARIAs (6). Furthermore, cerebrovascular comorbidities such as history of transient ischemic attack (TIA), symptomatic AIS, and a spontaneous intracranial hemorrhage not clearly explained by a prior condition expected to be self-limited without risk of recurrence (e.g., previously ruptured and obliterated intracranial aneurysm, traumatic subdural hematoma) were exclusionary (6, 7).

Anticoagulants are known to increase hemorrhagic risk in CAA. Interestingly, in Clarity AD, participants on anticoagulation were not excluded but indeed experienced a higher rate of cerebral macrohemorrhages (6). Consequently, fully anticoagulated patients are currently excluded from receiving ATTs. Although the absolute risk is uncertain, patients receiving ATTs may develop fatal intracerebral hemorrhage (ICH) after thrombolysis (e.g., in the setting of AIS) (8), which is therefore currently not recommended in these patients.

Even though patients with concomitant use of antiplatelets (single or dual) were not excluded from seminal RCTs of ATTs, their overly frequent—and oftentimes injudicious use in general population—may represent a potential risk for developing ARIA-H in individuals on ATTs (9–12). Moreover, a large, randomized trial in 19,114 healthy older adults showed a 38% increase in intracranial bleeding with low-dose aspirin (1.1 vs. 0.8%), without a statistically significant reduction in ischemic stroke, raising concerns about its role in primary prevention (13, 14).

Consequently, a meaningful clinical question arises about the safety of the concomitant antiplatelet treatment, especially DAPT, with regard to ARIA-H in individuals with AD on novel ATTs. This opinion paper discusses how the spectrum of medications with antiplatelet or platelet-modulating properties—beyond classical antithrombotics—may potentially influence ARIA and bleeding risk in ATT-treated patients, highlights current evidence and knowledge gaps, and considers future directions for research and clinical practice.

## 2 Discussion: possible drug-antibody interactions related to edema or hemorrhage

The risk landscape for amyloid-related imaging abnormalities (ARIA) and ICH in patients treated with anti-amyloid monoclonal antibodies extends beyond the established contributions of classical antiplatelet and anticoagulant agents. Key among these is a range of other medications frequently encountered in geriatric, neurological and psychiatric practice. A brief overview of these drug classes and their effects on hemostasis is presented in Table 1. Non-steroidal anti-inflammatory drugs (NSAIDs), for example, exert antiplatelet effects primarily through reversible inhibition of cyclooxygenase-1, diminishing thromboxane A2 production and therefore impairing platelet activation and aggregation (15). While the effect of NSAIDs on ARIA risk has not been systematically studied in the context of Alzheimer's disease treatments, extensive evidence links NSAID use to increased risk of systemic and, to a lesser degree, cerebral bleeding in older adults, especially when combined with other agents acting on hemostasis (16, 17).

**Table 1:** Medications with established or potential relevance to bleeding-risk assessment in patients considered for ATT.

Category	Drug class	Examples	Mechanism affecting hemostasis	Clinical considerations for ATT
Established risk (guideline-supported)	Antiplatelet agents	Aspirin, clopidogrel	COX-1 inhibition or P2Y12 blockade → reduced platelet aggregation	Use with caution; risk–benefit assessment required
	Dual antiplatelet therapy (DAPT)	Aspirin + P2Y12 inhibitor	Additive platelet inhibition	Typically avoided or requires deferral of ATT
	Anticoagulants	Warfarin, apixaban, rivaroxaban, dabigatran	Inhibition of coagulation factors	Generally excluded from ATT due to increased risk of ICH
Potential/modifying risk (limited or indirect evidence)	NSAIDs	Ibuprofen, naproxen, diclofenac	Reversible COX-1 inhibition → reduced platelet aggregation	Consider cumulative bleeding risk, especially with other agents
	SSRIs	Fluoxetine, sertraline, citalopram, etc.	Inhibition of platelet serotonin uptake → impaired aggregation	Possible additive bleeding risk; monitor in high-risk patients
	SNRIs	Venlafaxine, duloxetine	Partial serotonin-mediated platelet dysfunction	Likely lower risk than SSRIs; clinical significance uncertain
	Antipsychotics	Risperidone, clozapine, haloperidol	Variable effects on platelet receptors and thromboxane pathways	Limited evidence; individualized assessment recommended
	Antiepileptics	Valproic acid	Thrombocytopenia and impaired aggregation	Consider platelet count and bleeding history
Supplements (limited evidence)	Herbal supplements	Ginkgo biloba, garlic, turmeric	Inhibition of platelet aggregation	Clinical relevance uncertain; consider in comprehensive medication review
	Dietary supplements	Omega-3 fatty acids, vitamin E	Mild antiplatelet effects	No formal contraindication; caution in high-risk combinations

In addition to NSAIDs, other commonly prescribed drugs in patients with Alzheimer's disease that exhibit platelet-modulating activity include psychopharmacological treatments. Recent large-scale studies estimate that 30%−40% of eligible candidates for ATT are prescribed at least one psychiatric medication, and up to a quarter receive potentially problematic agents with pronounced antiaggregant activity (18). Among them, selective serotonin reuptake inhibitors (SSRIs) and, to a lesser degree, serotonin-norepinephrine reuptake inhibitors (SNRIs) are widely prescribed for depression and anxiety in older adults, including those with cognitive impairment. The principal mechanism underlying their bleeding risk is the inhibition of serotonin uptake into platelets via blockade of the serotonin transporter (SERT) on platelet membranes. Since platelets depend on serotonin for effective aggregation and hemostasis, SSRIs reduce intracellular serotonin, thereby impairing aggregation and increasing bleeding propensity (19, 20). This effect manifests most prominently as gastrointestinal bleeding, but several studies describe increased risks for intracranial hemorrhage as well (16, 21, 22). The magnitude of risk correlates with both the potency of SERT inhibition and cumulative exposure time; high-affinity SSRIs (fluoxetine, paroxetine, sertraline, citalopram, and escitalopram) pose greater risks compared to agents with weaker serotonergic activity (vortioxetine, trazodone, agomelatine etc.) (23). SNRIs (venlafaxine, duloxetine) may have a similar, though somewhat lower, risk profile for bleeding (24, 25). Importantly, the risk is additive with concomitant NSAIDs or antiplatelet agents and is potentially relevant for patients on amyloid-targeting monoclonal antibodies (ATTs) given their vulnerability to ARIA-H and cerebral amyloid angiopathy. Recent clinical guidelines and consensus statements on anti-amyloid therapy (26–28) explicitly acknowledge the importance of evaluating bleeding risk in Alzheimer's patients considered for ATT, especially in those co-prescribed antiplatelets or NSAIDs. While these guidelines advise individualized risk–benefit assessment and routine surveillance for amyloid-related imaging abnormalities (ARIA), detailed protocols for managing antidepressant or NSAID interactions remain limited, and the need for caution is emphasized in multimorbid and older populations.

Antipsychotic medications exert diverse effects on platelet function. Several antipsychotics, especially risperidone, clozapine, and haloperidol, have demonstrated inhibitory action on platelet aggregation via antagonism of purinergic P2Y1/P2Y12 receptors and disruption of the thromboxane A2 pathway (29). This theoretically confers antiplatelet activity and increased bleeding risk, but the real-world impact depends on drug choice, dose, and patient factors. In contrast, olanzapine and quetiapine (second-generation antipsychotics) have been reported to paradoxically activate platelets and increase prothrombotic risk, potentially contributing to vascular complications rather than bleeding (30). Data on the interaction of antipsychotics with ATTs and ARIA-H are lacking, and their mechanistic diversity necessitates individualized review in cognitive and psychiatric patient populations; however, in patients treated with AAT, the use of antipsychotics is generally less likely, as psychiatric complications in the early stages of Alzheimer's disease are not common.

Moreover, in several elderly patients, including those with dementia, antiepileptic drugs are used for epilepsy or as mood stabilizers, despite multiple guidelines advising against antiepileptic drugs, particularly valproic acid, for the treatment of behavioral or psychological symptoms of Alzheimer's disease due to poor efficacy and increased risk of adverse effects such as sedation, cognitive decline, and thrombocytopenia (31). Although valproate is now rarely initiated in early Alzheimer's disease, it remains used in real-world dementia care: recent US nursing-home data report valproic acid prescribing in approximately 12%−13% of residents with Alzheimer's disease and related dementias, particularly in those with disruptive behaviors (32). To our knowledge, specific data on concomitant valproic acid use in patients receiving amyloid-targeting monoclonal antibodies are not yet available, underscoring the need for dedicated pharmacovigilance in this subgroup. Valproic acid impairs platelet function by inhibiting aggregation and lowering platelet count, leading to clinically relevant bleeding risks (33), a key risk factor in anti-amyloid therapies. Most consensus guidance advocates avoiding anti-amyloid monoclonal antibodies in patients with severe thrombocytopenia or platelet dysfunction, but practical management of cases with borderline stable counts is infrequently addressed (5, 27, 34–38).

In regulatory and clinical safety guidance, lecanemab is reported to increase the risk of intracerebral hemorrhage when used concurrently with anticoagulant or thrombolytic agents (5, 39). European Medicines Agency documents and real-world cases have highlighted that additional caution is advised if thrombolytic agents (e.g., tissue plasminogen activators such as alteplase) are coadministered with lecanemab due to the plausible risk of severe intracranial bleeding (40). Although exposure to thrombolytic agents in clinical trials was limited, the risk of additive pharmacodynamic effects has been identified, particularly for anticoagulants (such as apixaban, warfarin, dabigatran, edoxaban, and rivaroxaban) and thrombolytics (such as alteplase and tenecteplase). Overall, no other clinically significant pharmacokinetic drug interactions have been established to date, but vigilance is warranted in patients requiring anticoagulation or thrombolytic therapy (27, 40).

In patients receiving one or more drugs or herbal/dietary supplements that increase the risk of bleeding (for example, low-dose aspirin, NSAIDs, or herbal preparations such as ginkgo biloba etc.), an individualized assessment of bleeding risk is essential when considering ATT. A thorough medication review can help optimize treatment and minimize the risk of bleeding (41–48).

### 2.1 Limitations of current evidence and knowledge gaps

The complex, multifactorial nature of polypharmacy in Alzheimer's disease management, where patients often receive combinations of analgesics, anticoagulants, psychotropics, and various disease-specific agents adds further unpredictability to bleeding risk and possible ARIA development (49). The presence of pharmacologically induced thrombocytopenia, whether from anticonvulsants (e.g., valproate), certain antibiotics (notably beta-lactams), or cancer therapies, may convert a subclinical ARIA to a clinically meaningful ICH. Polypharmacy-driven adverse interactions, such as serotonin depletion from SSRIs combined with platelet inhibition from NSAIDs or aspirin, highlight a matrix of risk that is cumulative, not simply additive (50). However, despite the plausible contribution of these drugs to adverse vascular events, data are currently insufficient to specify their risk in anti-amyloid antibody recipients, underscoring the need for more systematic pharmacovigilance and registries (51).

We also argue that it is important to highlight the current lack of knowledge on the specific risks associated with these potentially high-risk treatments in the context of CAA, as well as the fact that reliable detection of CAA remains suboptimal given present identification methods and diagnostic criteria, an issue with direct relevance for ATT.

Importantly, the current regulatory and consensus guidelines for anti-amyloid monoclonal antibodies in Alzheimer's disease, including lecanemab and donanemab, do not directly address these classes of non-antiplatelet, antiaggregant medications (5, 27, 34). Instead, the focus remains restricted to classical antithrombotic agents for eligibility and monitoring, overlooking drugs used routinely in the very populations most likely to be treated with disease-modifying therapies. Recent real-world eligibility studies and multicenter data analyses underscore both the magnitude and clinical consequences of these guideline omissions, raising the question of whether a more comprehensive approach to medication review and risk assessment is warranted in this evolving therapeutic landscape (18, 52, 53).

Given this context, we argue that multidisciplinary dialogue, combining neurological, psychiatric, geriatric, and pharmacy expertise may offer valuable perspectives in identifying individuals at elevated risk based on holistic medication profiles, not solely classic antithrombotics. Systematic data collection in registries, combined with post-marketing surveillance focused explicitly on polypharmacy and non-conventional antiaggregant medications, could further clarify the extent and clinical significance of these risks in routine practice.

## 3 Conclusions

As anti-amyloid monoclonal antibodies enter routine use for Alzheimer's disease, the narrow focus of current practice guidelines on classical antithrombotic agents overlooks a wider spectrum of medications that may significantly modulate bleeding and ARIA risk, including psychiatric drugs, NSAIDs, anticonvulsants, and common supplements. In our view, this gap risks underestimating real-world safety concerns for multimorbid patients almost certain to be exposed to polypharmacy. We strongly advocate for future consensus statements and eligibility criteria to explicitly broaden medication review beyond conventional antiplatelets and anticoagulants, incorporating individualized assessments of all agents with antiaggregant effects or bleeding risk. Until comprehensive data and guidance are available, we recommend interdisciplinary collaboration, detailed medication reconciliation, and vigilant imaging surveillance for all patients considered for amyloid-targeting therapies. In patients with an increased risk of bleeding or potential drug–drug interactions, a pharmacotherapeutic review by clinical pharmacists should be recommended before initiating therapy, to ensure treatment optimization and the safest possible use of the new medications. Addressing these complexities proactively will be essential to optimizing both safety and therapeutic benefit in Alzheimer's care.
